# High-energy extracorporeal shock wave therapy for nontraumatic osteonecrosis of the femoral head

**DOI:** 10.1186/s13018-017-0705-x

**Published:** 2018-02-02

**Authors:** Kai Xie, Yuanqing Mao, Xinhua Qu, Kerong Dai, Qingwei Jia, Zhenan Zhu, Mengning Yan

**Affiliations:** 1 Shanghai Key Laboratory of Orthopaedic Implants, Department of Orthopaedic Surgery, Shanghai Ninth People’s Hospital, Shanghai Jiao Tong University School of Medicine, Shanghai, China; 2 Affiliated Hospital of Taishan Medical University, Taian, China

**Keywords:** High-energy extracorporeal shock wave therapy, Osteonecrosis, Femoral head, Follow-up studies, Bone marrow edema

## Abstract

**Background:**

Nontraumatic osteonecrosis of the femoral head (ONFH) is treated with a series of methods. High-energy extracorporeal shock wave therapy (ESWT) is an option with promising mid-term outcomes. The objective of this study was to determine the long-term outcomes of ESWT for ONFH.

**Methods:**

Fifty-three hips in 39 consecutive patients were treated with ESWT in our hospital between January 2005 and July 2006. Forty-four hips in 31 patients with stage I–III nontraumatic ONFH, according to the Association Research Circulation Osseous (ARCO) system, were reviewed in the current retrospective study. The visual analog pain scale (VAS), Harris hip score, radiography, and magnetic resonance imaging were used to estimate treatment results. The progression of ONFH was evaluated by imaging examination and clinical outcomes. The results were classified as clinical success (no progression of hip symptoms) and imaging success (no progression of stage or substage on radiography and MRI).

**Results:**

The mean follow-up duration was 130.6 months (range, 121 to 138 months). The mean VAS decreased from 3.8 before ESWT to 2.2 points at the 10-year follow-up (*p* < 0.001). The mean Harris hip score improved from 77.4 before ESWT to 86.9 points at the 10-year follow-up. The clinical success rates were 87.5% in ARCO stage I patients, 71.4% in ARCO stage II patients, and 75.0% in ARCO stage III patients. Imaging success was observed in all stage I hips, 64.3% of stage II hips, and 12.5% of stage III hips. Seventeen hips showed progression of the ARCO stage/substage on imaging examination. Eight hips showed femoral head collapse at the 10-year follow-up. Four hips in ARCO stage III and one hip in ARCO stage II were treated with total hip arthroplasty during the follow-up. Three were performed 1 year after ESWT, one at 2 years, and one at 5 years.

**Conclusions:**

The results of the current study indicated that ESWT is an effective treatment method for nontraumatic ONFH, resulting in pain relief and function restoration, especially for patients with ARCO stage I–II ONFH.

## Background

Osteonecrosis of the femoral head (ONFH) is mainly associated with significant hip pain and dysfunction in young adults. ONFH was reported to affect 20,000 patients each year in America [1]. The estimated yearly incidence of ONFH in Korea is 37.96/100000 [2]. Most patients without an effective treatment in the early stage require hip joint replacement. About 49% of untreated asymptomatic ONFH hips progressed to collapse at 49 months following diagnosis [3]. Postcollapse ONFH is one of the most common reasons for primary total hip arthroplasty in many countries [2, 4]. Given the relatively young age at the time of presentation, it is reasonable to preserve the native hip in patients with early-stage ONFH. Several different joint-preserving operative interventions have been reported with promising outcomes in the past decades, including core decompression, osteotomy, and vascularized/nonvascularized bone grafting.

Unlike those operative interventions, biophysical therapy is considered as a noninvasive method for ONFH treatment [5]. Biophysical techniques such as extracorporeal shock wave therapy (ESWT) have also been reported to enhance bone formation and preserve the femoral head in osteonecrosis [6, 7]. The use of ESWT in the field of orthopedics started in the 1990s, and the main indications were calcified tendonitis, heel pain, and fracture nonunion [8]. In 2001, the first report on ESWT for ONFH showing promising short-term results was published [6]. Subsequently, a randomized controlled trial showed that ESWT was more effective than core decompression and nonvascularized bone grafting for early-stage ONFH treatment [7]. Some good-to-excellent outcomes in pain relief, functional improvement, and hip survival have been reported in the past decade [9–13]. However, the long-term outcomes of ESWT for ONFH remain unknown. Therefore, the objective of this study was to determine the long-term outcomes of ESWT for ONFH.

## Methods

The current retrospective study was approved by our institutional review board. Fifty-three hips in 39 consecutive patients underwent ESWT in our hospital between January 2005 and July 2006. Written informed consent was obtained for each patient who participated in the current study according to our institutional policy. The diagnosis of nontraumatic ONFH was based on history, clinical examination, and imaging assessment. The inclusion criteria were patients with symptomatic early-stage ONFH, which was defined as Association Research Circulation Osseous (ARCO) stages I–III ONFH with hip pain and/or dysfunction (Table 1), [14, 15]. The exclusion criteria were ONFH patients with the following: (1) former surgical treatment, (2) history of hip trauma, and (3) ARCO stage IV.

**Table 1:** Association Research Circulation Osseous classification of osteonecrosis [14, 15]

Stage	Findings	Subclassification	Quantitation
0	All present techniques normal	No	No
I	Radiography and computed tomography normal; at least one of the other techniques is positive	Location of lesion Medial Central Lateral	Area of involvement (%) A: < 15% B: 15 to 30% C: > 30% Length of crescent A: < 15% B: 15% to 30% C: > 30% Surface collapse and dome depression A: < 15% and < 2 mm B: 15% to 30% and 2 mm to 4 mm C: > 30% and > 4 mm
II	No crescent sign; Sclerosis, osteolysis, focal porosis		
III	Crescent sign and/or flattening of articular surface		
IV	Osteoarthritis, joint space narrowing, acetabular changes, joint destruction	No	No

The mean follow-up duration was 130.6 months (range, 121 to 138 months). Five hips in five patients with traumatic ONFH and two hips in one patient with ARCO stage IV ONFH were excluded. Two patients (two hips) were unable to participate in the current study due to personal reasons. Forty-four symptomatic nontraumatic ONFH hips in 31 patients (32 hips in 23 male patients and 12 hips in 8 female patients) with a mean age of 41.2 years (range, 22 to 60 years) were included in the current study (Table 2). Sixteen patients with 24 hips were on a high-dose of corticosteroids. Seven patients with nine hips had a history of alcohol abuse. Eleven hips in eight patients with no established risk factor were considered as having idiopathic ONFH. According to the ARCO classification, eight hips were stage I, 28 hips were stage II, and the remaining were stage III.

**Table 2:** Characteristics of patients

Characteristic	Total
Number of patients	31
Number of hips	44
Sex	
Male	23
Female	8
Mean age (year)	41.2
Mean follow-up (month)	130.6
ARCO stage (hips)	
I	8
II	28
III	8
Risk factor (hips)	
Corticosteroid	24
Alcoholic	9
Idiopathic	11

ESWT was performed by two senior doctors, under spinal anesthesia or general anesthesia. The patients were placed in the supine position on the radioparent operation table with the limbs secured to the table. The femoral artery was identified and marked on the skin to avoid direct shock during the procession of treatment. The lesion on the femoral head was identified using a C-arm (Siemens, Germany) in ARCO stage II and III patients before treatment. In stage I patients, the lesion was identified according to MRI. Four focal points were selected around the lesion under the C-arm to receive extracorporeal shock wave therapy with an OssaTron (HMT, Switzerland). Each point was treated with 1000 impulses of shock waves at 26 kV and 4 Hz. Treatment was performed bilaterally in 13 patients, and all hips received a single treatment. After treatment, the patient was asked to ensure strict no weight-bearing to limited weight-bearing in the first 3 months; full weight-bearing was allowed at 3 months postoperatively.

The visual analog pain scale (VAS), Harris hip score, and the radiography and magnetic resonance imaging (MRI) scans were collected before treatment and during the follow-up. Hip function was evaluated using the Harris hip score. The VAS was used to evaluate pain relief after treatment. Imaging examinations including standardized radiography and MRI were performed to evaluate the ARCO stage of the disease and the bone marrow edema (BME) of the femoral head. According to the range of edema, BME is divided into five grades: grade 0 for no BME, grade 1 for peri-necrotic BME, grade 2 for BME extending into the femoral head, grade 3 for BME extending into the neck of the femur, and grade 4 for BME extending into the intertrochanteric region [10]. The results were classified as a clinical success (no progression of hip symptoms), an imaging success (no progression of stage or substage on the radiography and MRI), or failure (progression of hip symptoms or ARCO stage).

SAS version 8.0 (SAS Institute Inc., USA) was used to perform all statistical calculations. The outcomes at the final follow-up were compared with data before ESWT using the *t* test. A two-tailed *p* value of less than 0.05 was considered significant.

## Results

The mean Harris hip score improved significantly from 77.4 before ESWT to 86.9 points at the 10-year follow-up (*p* < 0.001). The mean VAS score decreased significantly from 3.8 preoperatively to 2.2 points at the final follow-up (*p* < 0.001). The outcomes of ESWT were different in patients with different ARCO stages and pathogeny at the final follow-up (Table 3). The clinical success was defined as no progression of hip symptoms, which was observed in 87.5% of ARCO stage I patients, 71.4% of ARCO stage II patients, and 75.0% of ARCO stage III patients. ESWT was most effective in patients with idiopathic ONFH. Four hips in ARCO stage III and one hip in ARCO stage II underwent total hip arthroplasty (THA) during the follow-up, because of aggravated disease with unacceptable pain and hip dysfunction. Three (two hips in ARCO stage IV and one hip in ARCO stage IIIc) underwent THA 1 year after ESWT, one (ARCO stage IV) at 2 years, and one (ARCO stage IV) at 5 years.

**Table 3:** Clinical outcome of patients with different ARCO stage and risk factor

	Before ESWT	Final follow-up	*P* value
Total			
Harris hip score	77.4 ± 15.1	86.9 ± 13.7	< 0.001
VAS	3.8 ± 2.6	2.2 ± 2.4	< 0.001
ARCO stage			
ARCO stage I			
Harris hip score	84.9 ± 12.4	96.6 ± 4.0	0.033
VAS	2.9 ± 2.2	0.5 ± 0.8	0.015
ARCO stage II			
Harris hip score	80.2 ± 14.1	88.9 ± 11.9	0.005
VAS	3.3 ± 2.6	1.8 ± 2.1	0.008
ARCO stage III			
Harris hip score	59.9 ± 6.7	70.2 ± 12.7	0.083
VAS	6.5 ± 0.9	4.9 ± 2.5	0.155
Risk factor			
Corticosteroid			
Harris hip score	79.8 ± 15.5	87.2 ± 12.9	0.020
VAS	3.5 ± 2.7	2.4 ± 2.5	0.063
Alcoholic			
Harris hip score	72.0 ± 19.0	78.4 ± 15.8	0.198
VAS	4.4 ± 2.8	3.4 ± 2.5	0.201
Idiopathic			
Harris hip score	76.5 ± 10.3	93.3 ± 10.9	0.001
VAS	4.0 ± 2.1	0.7 ± 0.8	< 0.001

**Fig. 1:**
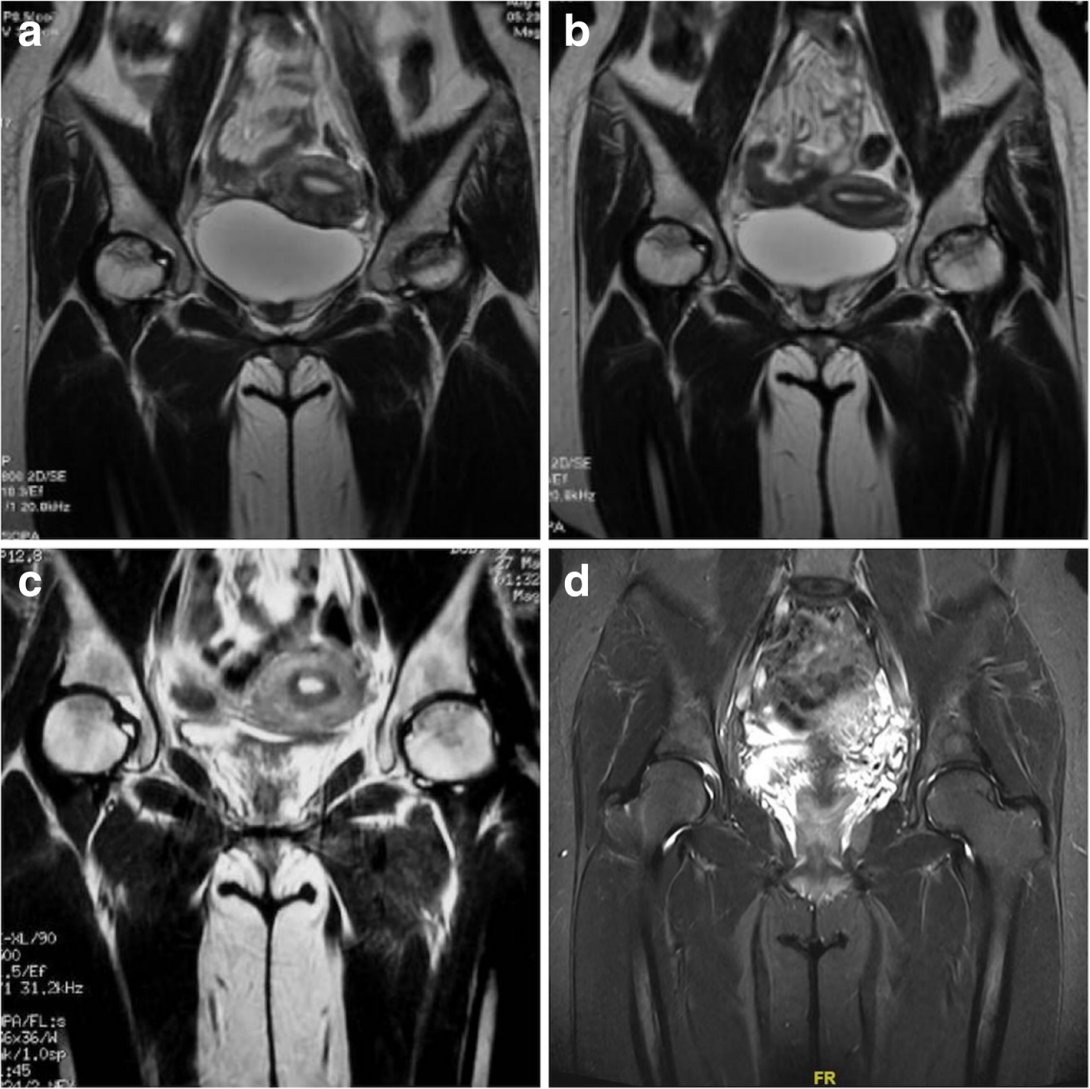
MRI of a young woman with high-dose corticosteroid use: **a** MRI indicated bilaterally ARCO stage II ONFH before ESWT; **b** MRI in 3 months after ESWT; **c** MRI in 5 years after ESWT; **d** No lesion was observed in MRI at final follow-up. The patient has fully hip function without pain at the final follow-up

**Fig. 2:**
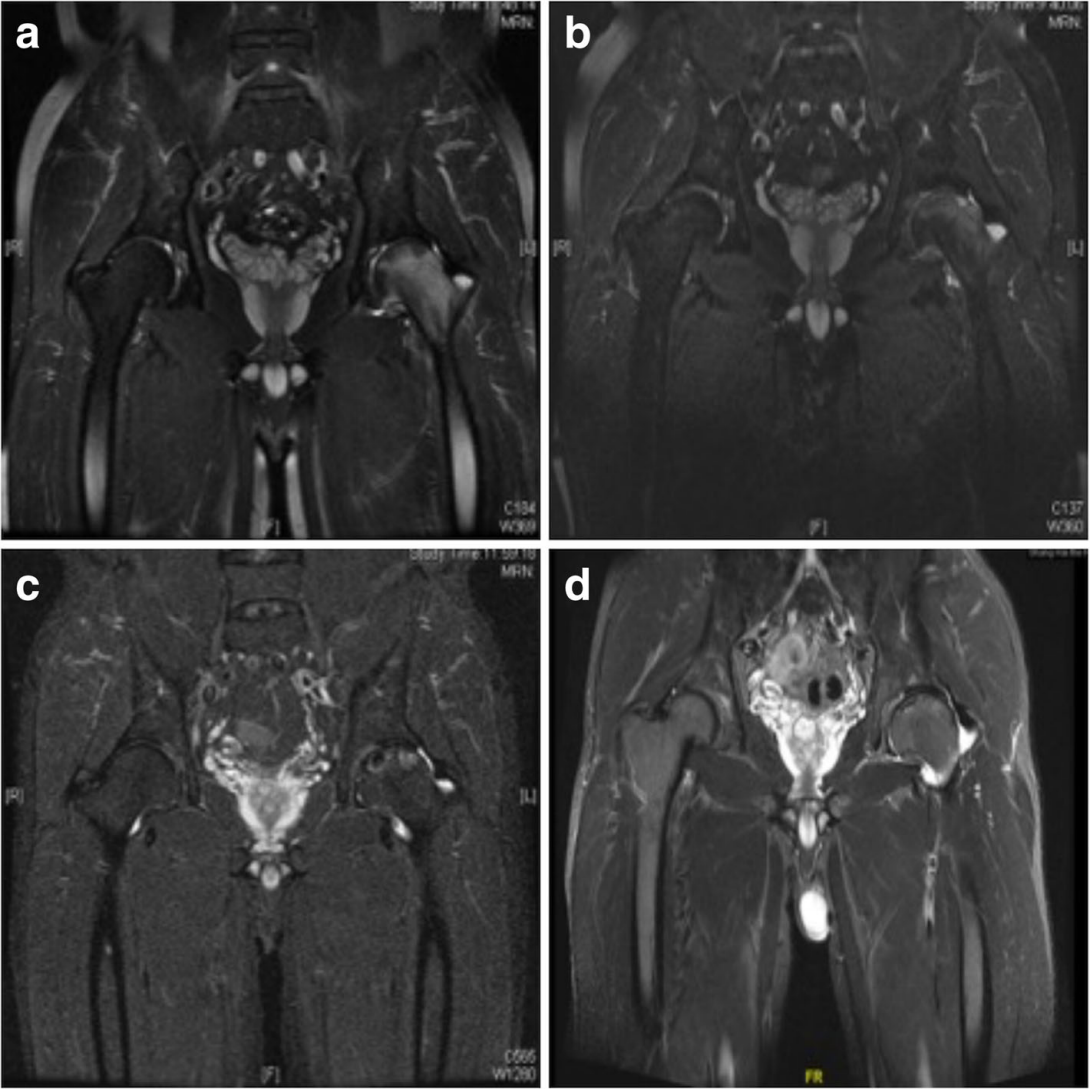
MRI of a mid-age man with long-term alcohol abuse: **a** MRI indicated ARCO stage II ONFH with grade 4 BME in the left hip before ESWT; **b** BME reduction was observed in 3 months after ESWT; **c** MRI in 5 years after ESWT; **d** MRI indicated grade 2 BME at the final follow-up. The patient has restored hip function without pain since 5 months after ESWT

All patients received imaging examinations before treatment and at the follow-up. Imaging success was observed in all stage I hips, 64.3% of stage II hips, and 12.5% of stage III hips. At the last follow-up, lesions in three stage I hips and one stage II hip could not be detected on MRI (Fig. 1). A total of 17 hips showed progression of the ARCO stage/substage on radiography or MRI. At the last follow-up, eight hips showed femoral head collapse on standardized radiographs (Table 4). Five of them received THA during the follow-up; the three remaining patients used non-steroidal anti-inflammatory drugs to reduce hip pain. BME around the focal osteonecrosis was observed on MRI before ESWT in all hips included in the current study. Ten hips had grade 1 BME, 6 hips had grade 2 BME, 13 hips had grade 3 BME, and 15 hips had grade 4 BME before ESWT. A reduction in BME was also noted in 30 hips at the final follow-up. Thirteen hips showed no significant change in BME at the final follow-up (Fig. 2). Only one ARCO stage II hip with grade 2 BME progressed to grade 3. In patients with improved BME, the mean VAS score was 1.6 points at the 10-year follow-up, and the mean Harris hip score was 89.7 points at the 10-year follow-up. In patients with unchanged BME, the mean VAS score was 3.5 points and the mean Harris hip score was 79.8 points at the last follow-up. Clinical outcomes were better in the BME improved group than in the BME unchanged group (Table 5).

**Table 4:** Change in MRI before ESWT and at 10-year follow-up

	Improved	Unchanged	Progressed	Collapsed
ARCO stage I				
Ia	2	5	0	0
Ib	1	0	0	0
Ic	0	0	0	0
Total	3	5	0	0
ARCO stage II				
IIa	0	1	1	0
IIb	6	3	3	1
IIc	5	3	6	2
Total	11	7	10	3
ARCO stage III				
IIIa	0	1	3	1
IIIb	0	0	1	1
IIIc	0	0	3	3
Total	0	1	7	5

**Table 5:** Clinical outcome of patients with different BME change

	Before ESWT	Final follow-up	*P* value
Harris hip score			
BME improved			
Total	77.8 ± 15.6	89.7 ± 11.1	< 0.001
ARCO stage I	84.6 ± 15.4	95.6 ± 4.5	0.175
ARCO stage II	80.5 ± 14.3	91.1 ± 11.0	0.006
ARCO stage III	60.4 ± 8.5	78.0 ± 9.1	0.045
BME unchanged			
Total	77.2 ± 15.1	79.8 ± 17.2	0.354
ARCO stage I	85.3 ± 8.1	98.3 ± 9.1	0.157
ARCO stage II	81.4 ± 14.5	81.6 ± 12.8	0.970
ARCO stage III	59.0 ± 3.5	57.3 ± 1.2	0.560
BME progressed			
Total	67.0	96.0	/
ARCO stage II	67.0	96.0	/
VAS			
BME improved			
Total	3.8 ± 2.8	1.6 ± 1.8	< 0.001
ARCO stage I	3.1 ± 2.7	0.8 ± 0.8	0.118
ARCO stage II	3.3 ± 2.8	1.5 ± 1.9	0.005
ARCO stage III	6.5 ± 1.1	3.3 ± 1.2	0.019
BME unchanged			
Total	3.7 ± 2.2	3.5 ± 3.2	0.734
ARCO stage I	2.5 ± 1.5	0 ± 0	0.102
ARCO stage II	3.0 ± 1.9	3.1 ± 2.4	0.873
ARCO stage III	6.4 ± 0.4	7.6 ± 0.6	0.148
BME progressed			
Total	5.5	1.5	/
ARCO stage II	5.5	1.5	/

## Discussion

The current study showed that significant improvements in pain relief and function restoration were maintained for more than 10 years after ESWT. The mean Harris hip score improved from 77.4 before ESWT to 86.9 points at the final follow-up. The mean VAS score decreased from 3.8 preoperatively to 2.2 points at the 10-year follow-up. Four ARCO stage III hips and one ARCO stage II hip underwent THA during the follow-up due to unacceptable pain and hip dysfunction. The improvement of clinical assessments in our study was comparable with those in former short and mid-term reports [6, 7, 9–12]. In patients with clinical success, pain relief and functional restoration often occurred between 3 months and 1 year after ESWT and were maintained for more than 10 years. The exacerbation of symptoms would appear at 5 months to 10 years after treatment. We consider it necessary to evaluate the affected hip once every year after ESWT.

According to imaging assessments, 14 hips (31.8%) showed improved images with decreased lesion size, and 13 hips (29.5%) showed no significant change in ARCO stage/substage. Seventeen hips (38.6%) showed progression of the ARCO stage, and eight hips (18.1%) showed femoral head collapse on standardized radiographs at the last follow-up. According to imaging assessments, ESWT could prevent progression of the disease in ARCO stage I and II hips. For ARCO stage III hips, a significant progression of the disease was observed during the follow-up. A significant reduction in BME was also noted in most hips at the 10-year follow-up. BME of the proximal femur could be commonly detected by MRI in patients with symptomatic ONFH. BME could increase the bone marrow pressure, which may reduce the blood supply and promote avascular necrosis of the femoral head [16]. A former study by Koo et al. showed that BME of the proximal femur was strongly related to joint pain in patients with early-stage ONFH [17]. Huang et al. analyzed radiograph and MRI scans of 71 ONFH patients and found that 98% of osteonecrotic hips with BME were painful [18]. ESWT has been reported to be effective for the treatment of BME in ONFH patients [9, 10]. In our study, the clinical outcomes in patients with BME reduction are superior to those in other patients, which indicated that physical decompression caused by BME reduction is beneficial to ONFH patients.

The aim of early-stage ONFH treatment is to prevent collapse by delaying the natural progression of the disease. Several joint-preserving operative interventions have been used in the past decade. Given their unpredictable long-term clinical outcomes, none of these methods is generally optimal. Core decompression is the most common joint-preserving operation for early-stage ONFH treatment worldwide. However, several systematic reviews and meta-analyses indicated that core decompression did not provide a significant difference in the collapse rate when compared with other joint-preserving treatments [19–21]. Wang et al. compared the outcome between ESWT and core decompression with bone grafting. ESWT had better clinical outcomes in terms of pain relief, function restoration, and THA rate when compared with core decompression [9]. The present study findings suggest promising long-term results of ESWT for early-stage ONFH.

Several studies also reported that ESWT was more effective in early-stage ONFH (ARCO stage I and II) [7, 10, 11]. Our study confirmed that ARCO stage III patients benefit less from ESWT than ARCO stage I and II patients. The 10-year survival of ARCO stage III hips was 50%, which was also inferior to that of other groups. Based on the information now available, we suggest that further randomized controlled trial studies should be performed to confirm the effectiveness of ESWT for patients with ARCO stage I and II ONFH.

A former study analyzed the outcome of ESWT for ONFH in systemic lupus erythematosus (SLE) patients with corticosteroid use [22]. Both SLE and non-SLE patients showed a significant improvement in the clinical outcome and imaging studies, and no statistically significant differences were observed between the two groups. Because of the limited number of patients, we are still unable to determine whether different pathogenies could affect the treatment outcome of ESWT. The mean Harris hip score (78.4) and mean VAS score (3.4) in patients with alcohol abuse were inferior to those in patients with corticosteroid-related and idiopathic ONFH at the final follow-up. However, more than half (five of nine hips) of the alcoholic patients had ARCO stage III hips. As we mentioned above, ARCO stage III ONFH is often associated with a poor outcome after ESWT.

As a non-invasive treatment method, ESWT has been reported as an effective treatment method for musculoskeletal diseases since the 1990s. However, the true treatment mechanism of ESWT for pain relief and tissue remolding has not been fully understood. Wang et al. reported that ESWT promoted bone healing by increasing neovessels and upregulated growth factors at the tendon-bone junction [23]. Immunohistochemical examination indicated that ESWT upregulates the expression of vWF, VEGF, and CD31 in the human femoral head [24]. Localized hematoma and cell death caused by direct shock could also promote new bone formation [25]. Several animal studies indicated that the pain relief with ESWT could be owing to diminished pain transmission to the central nervous system. The stimulation of the extracorporeal shock wave to the distal femur could decrease the release of substance P after 6 weeks in rabbits [26]. Moreover, in the dorsal root ganglion of rabbits, neurons immunoreactive for substance P were depressed after extracorporeal shock wave treatment to the distal femur [27]. Based on our results, we hypothesize that the significant pain relief in ONFH patients after ESWT is based on BME reduction. The physical decompression caused by BME reduction would increase the blood supply in focal lesions.

The current study has several limitations that should be acknowledged. First, the current study is limited by its retrospective design. Second, the limited number of participants may have caused bias in the assessment of the outcomes. Third, there was no control group. A comparison between ESWT and other treatments would be useful to determine the superiority of ESWT in the treatment of ONFH in well-selected patients.

## Conclusions

In summary, ESWT is an effective treatment method for early-stage nontraumatic ONFH. Significant improvements including pain relief and functional restoration were maintained for more than 10 years after treatment. More large-scale randomized controlled trial studies should be performed to confirm the effectiveness of ESWT for early-stage nontraumatic ONFH.

## Abbreviations

ARCO: Association Research Circulation Osseous
BME: Bone marrow edema
ESWT: High-energy extracorporeal shock wave therapy
MRI: Magnetic resonance imaging
ONFH: Osteonecrosis of the femoral head
SLE: Systemic lupus erythematosus
VAS: Visual analog pain scale
